# The Influence of Masculinity and the Moderating Role of Religion on the Workplace Well-Being of Factory Workers in China

**DOI:** 10.3390/ijerph18126250

**Published:** 2021-06-09

**Authors:** Quan Gao, Orlando Woods, Xiaomei Cai

**Affiliations:** 1 School of Social Science, Singapore Management University, Singapore 178903, Singapore; qgao@smu.edu.sg (Q.G.); orlandowoods@smu.edu.sg (O.W.); 2 School of Tourism Management, South China Normal University, Guangzhou 510000, China

**Keywords:** masculinity, religion, health, workplace well-being, factory workers

## Abstract

This paper explores how the intersection of masculinity and religion shapes workplace well-being by focusing on Christianity and the social construction of masculinity among factory workers in a city in China. While existing work on public and occupational health has respectively acknowledged masculinity’s influences on health and the religious and spiritual dimensions of well-being, there have been limited efforts to examine how variegated, and especially religious, masculinities influence people’s well-being in the workplace. Drawing on ethnography and in-depth interviews with 52 factory workers and 8 church leaders and factory managers, we found that: (1) Variegated masculinities were integrated into the factory labor regime to produce docile and productive bodies of workers. In particular, the militarized and masculine cultures in China’s factories largely deprived workers of their dignity and undermined their well-being. These toxic masculinities were associated with workers’ depression and suicidal behavior. (2) Christianity not only provided social and spiritual support for vulnerable factory workers, but also enabled them to construct a morally superior Christian manhood that phytologically empowered them and enhanced their resilience to exploitation. This paper highlights not only the gender mechanism of well-being, but also the ways religion mediates the social-psychological construction of masculinity.

## 1. Introduction

Since 2010, more than 10 young male factory workers have committed suicide at an electronics manufacturing company located in China. These suicide events not only exposed the notoriously militaristic, masculine, and highly exploitative factory management system but also focused world-wide attention onto the psychological well-being of Chinese factory workers. Importantly, whilst the suicides frequently happened amongst men, the assembly line work was stereotypically considered a women’s job [1,2]. With this in mind, the gender politics of masculinity underlying factory workers’ suicidal behavior warrants closer examination.

Complicating the gendering of work and suicide in China is the role of social welfare organizations, especially religious institutions, therein. Indeed, in response to the suicide events since 2010, a large number of Christian institutions (including churches and faith-based organizations) emerged to provide social support and spiritual care for factory workers. Existing research has suggested that Christianity plays an important role in shaping working-class cultures and in mediating labor relations in the workplace [3,4]. Factory workers draw on Christian ethics and worldviews to navigate labor exploitation, thus reframing their experiences of marginality. In short, then, the phenomenon of factory workers in China provides a social niche to examine the intersections of masculinity, religion and workplace well-being.

Existing literature on public and occupational health reflects a growing interest in the gender dynamics of health and specifically how masculinity influences people’s health practices and well-being [5,6,7]. Many studies show that hegemonic masculinity can undermine people’s psychological well-being by reproducing dominant gender relations (e.g., [5]). In particular, hegemonic masculinity contributes to fostering an unhealthy workplace culture that encourages power struggles and toxic leadership [8,9]. Nevertheless, there are two areas of enquiry that have not been fully explored through this line of research. First, research needs to pay attention to the variegated forms of masculinities and their diverse influences on workplace well-being. Second, religion is largely absent from the literature on masculinity and health, although the influences of religion and spirituality on well-being and resilience have been recognized elsewhere [10,11,12]. This paper therefore fills the lacuna by examining the interlocking relations of masculinity, religion and well-being through a ethnographic study of Christian factory workers in China. Moreover, this paper highlights not only the gender mechanisms of well-being within China’s factories but also the how religion mediates the social-psychological constructions of masculinity.

## 2. Incorporating Masculinity and Religion into Workplace Well-Being

### 2.1. Masculinities and Well-Being in the Workplace

Over the past two decades or so, researchers of gender and health have exhibited growing interest in how constructions of masculinity influence people’s health and well-being [5,6,7,13,14,15]. This interest builds on early research from feminist health scholars, which suggested that health practices are gendered. Women are far more interested in health-promotion than men, and have healthier lifestyle patterns as a result, while men engage less in self-care, especially the search for medical assistance [16,17]. However, Courtenay criticizes the fact that research on “gender and health” has simply treated gender as an analytical category, and has therefore become largely synonymous with research of “gender difference of health” [5]. Instead, health scholars’ growing interest in masculinity signifies a shift in the focus of feminist health studies: from gender difference, to how the relationalities and power of gender shapes health and well-being. In essence, attending to masculinity does not simply provide a perspective for exploring men’s idiosyncratic health and well-being practices, but also opens up a relational approach to examining how health practices are co-produced and mediated by relations between men and women, and between different men themselves [18]. For example, Courtenay argues that “a relational theory of men’s health” would examine “how men construct various forms of masculinity (or masculinities) and how these different enactments of gender, as well as differing social structural influences, contribute to” different health subjectivities and practices [5] (p. 1387). In this formulation, some scholars suggest that notions like “hegemonic masculinity” [18] and “protested masculinity” [19] are particularly helpful to understanding the gender dynamics of health and well-being.

The concept of hegemonic masculinity is one of the most influential contributions to gender studies, and provides a useful analytical framework for examining patriarchy and gendered hierarchies, labor and class relations [18,20]. Connell and Messerschmidt [18] (p. 832) define hegemonic masculinity as the hegemonic discourses and normative pattern of being a man, which “require[s] all other men to position themselves in relation to it, and it ideologically legitimate[s] the global subordination of women to men”. “Hegemony” in this context means the exercise of power and those who have the ability to dictate which forms of masculinities are desired and idealized so that they appear “normal” and “natural”. Hegemonic masculinities are often normalized by culturally and economically privileged men (e.g., white, heterosexual, middle-class men), which contrast to gender practices held by less powerful groups, such as the marginalized, or the protested masculinities of black and working-class men [20]. As Gao suggests, “protested masculinities” or “marginal masculinities” can be understood as a form of gender practice that “marginalized men utilize to open a dialogical space with mainstream society and state power through deviant behaviors such as being tough, rebellious and violent, thereby restoring their lack of power or maintaining their cultural, regional or ethnic privilege” [21] (p. 288). However, even working-class men themselves can express hegemonic masculinity in order to maintain a normative working-class image. In this sense, as Connell and Messerschmidt [18] note, hegemonic masculinity is always relationally defined and constructed in particular cultures and contexts.

Some important studies have suggested that hegemonic masculinities are harmful to men’s health and well-being (e.g., [5,22]). Specifically, the power struggles inflicted by hegemonic masculinity often lead to men harming themselves:

The social practices that undermine men’s health are often the instruments men use in the structuring and acquisition of power. Men’s acquisition of power requires, for example, that men suppress their needs and refuse to admit to or acknowledge their pain. Additional health-related beliefs and behaviors that can be used in the demonstration of hegemonic masculinity include the denial of weakness or vulnerability, emotional and physical control, the appearance of being strong and robust, dismissal of any need for help, a ceaseless interest in sex, the display of aggressive behavior and physical dominance [5] (p. 1389, emphasis in original).

Moreover, hegemonic masculinity is shown to be associated with men’s depression and therefore increases the risk of suicidal behaviors [22]. Clinical psychologists have revealed that depression is incompatible with some hegemonic forms of masculinities, because [23]: (1) depression, with its emotional manifestation, is linked to femininity; and (2) depression is believed to be an indication of powerlessness, lack of control and vulnerability, which is at odds with the cultural representation of masculinity as being tough and self-reliant. In a study that examined men’s account of depression, Emslie et al. [22] also demonstrated how the pressures from conforming to hegemonic masculinity could lead to a higher level of suicidal behavior among men. However, they also revealed that “a minority of men had found ways of being masculine which were outside hegemonic discourses” and therefore escaped from the trap of hegemonic masculinity that constrained them [22] (p. 2246). Similarly, Kazmierczyk et al.’s study of mobbing at the workplace also showed that men are more often the victims of mobbing/bullying as compared to women, as “men cope worse with criticism or a lack of appreciation” [24] (p. 710).

It is noteworthy that these unhealthy gender practices are often reproduced by particular institutional structures (e.g., the government and the military) in which “unhealthy beliefs and behaviors among men” [5] (p. 1394) are fostered. Recently, there is an emerging line of research suggesting that the workplace is such a structure in which hegemonic masculinity persists, and can undergird a culture that undermines people’s well-being [8,9]. For example, a recent special issue in the Journal of Social Issues focused on the “masculinity contest cultures” in the workplace, and explored the association between hegemonic masculinity and the “norms, rituals, and belief systems valorizing social dominance, work above other parts of life, physical strength” [9] (p. 500). This collection shows that masculinity contest cultures could bring about higher stress, exacerbate work/life conflicts and thwart the positive meaning of work. In particular, Matos, O’Neill and Lei [9] demonstrated that hegemonic masculinity could breed toxic forms of leadership in the workplace, or a leadership style characterized by abusive behaviors that are used to bully, control and exploit others [25]. Toxic leadership therefore produces a “Dog-Eat-Dog” atmosphere at the workplace, which encourages unhealthy competition and a desire for authority over others [9].

Although existing research has indicated a strong link between masculinity and workplace cultures, relatively less concern has been paid to the ways that masculinity influences workplace health and well-being. In particular, how variegated forms of masculinities (hegemonic, protested, marginal, or others) contest and shape one another in the workplace, and how they could impact on people’s well-being differently, are still underexplored. Religion is one of the factors that may create alternative definitions of masculinity in the workplace, and therefore requires further analysis.

### 2.2. Religious Masculinity and Well-Being

Religion is largely absent from the literature exploring the intersections of masculinity and health. We argue that the lack of engagement in religion’s influences on masculinity and health leads to a failure to address not only the spiritual dimensions of health, but also the variegated forms of masculinity. This paper therefore attempts to bring masculinity, religion and health into conservation with one another in order to understand the mediating role of religion in the gender dynamics of health.

On the one hand, some research has suggested that masculinity is often religiously constituted and mediated [21,26,27,28]. Religion provides a cultural, theological and ethical framework through which masculinity is constructed and performed. For example, in Western cultures, Muslim masculinity is often represented in the dominant imagination as violently patriarchal and aggressive [26]. This image may be influenced by public (mis)understandings of the discourse of jihad [28]. However, this stereotype of Muslim masculinity does not capture the piety and the “actually existing” religious practices performed in the everyday life by most Muslim men, and thus places them in a culturally marginalized position in Western society [26]. However, compared with Muslim masculinities, masculinities of Christian men have received scant academic attention. Some theological and biblical scholars have argued that Christian masculinity is characterized by the performance of Christian virtues, and in particular, the spiritual ability to control one’s passions, desires and angers [29,30]. In particular, the Christian discourse of men’s headship requires men to take more responsibility than women, although this form of masculinity is itself hegemonic [31]. Gallagher and Smith [31] argue that Christian masculinity is better understood as a “soft patriarchy” that highlights men’s spiritual privilege in front of God, but simultaneously emphasizes their love and responsibility towards their wives. Brusco’s [32] study of an evangelical movement in Latin America found that Christian men’s masculinity was linked to asceticism, thus helping men maintain a more self-disciplined lifestyle by encouraging them to drink, smoke and gamble less. Nevertheless, religious masculinities are enacted by particular teachings and beliefs, but are better understood as a cultural strategy that men use to negotiate the social-cultural environment around them.

On the other hand, research has shown that religion and spirituality play an important role in shaping people’s health practices and psychological well-being (e.g., [10,11,12]). For example, based on a quantitative analysis of U.S. citizens, Miller and Thoresen showed a positive relationship between religiousness and citizens’ well-being, although the rationale behind such a relationship remains unclear [12]. Religion and spirituality have been found to be important factors in enhancing patients’ ability to cope with chronic and serious illness, depression, and mental health [33,34,35]. In particular, many researchers use the concept of “resilience” to understand religion’s influences on health. Spirituality is shown to be an important source of resilience, providing the ability and capacity to recover from difficult life events and depression, or other psychological vulnerabilities [36]. Some research has indicated that religion and spirituality enhance people’s resilience by providing a source of comfort and hope, as well as an alternative worldview to make sense of the adversity around them [36,37]. In general, health is not simply a bio-physical process, but “is powerfully influenced by cultural, social, and philosophical factors, including the existence of meaning and purpose in life and the quality of intimate personal relationships” [12] (p. 25). It is essential, therefore, that we find variegated pathways to resilience and psychological well-being, and explore the mechanisms that shape the social, cultural and psychological processes of resilience.

However, although religion’s influences on masculinity and health have been acknowledged, how the intersection of religion and masculinity can shape people’s well-being and resilience is still unclear. Above all, this paper fills the lacuna by examining: (1) the variegated and contested masculinities at the workplace and their impacts on workers’ well-being; and (2) how religion creates new masculinities, and how this religious masculinity influences well-being and the resilience of workers.

## 3. Materials and Methods

### 3.1. Research Contexts and Research Questions

We chose China’s factories and especially Company A as our research setting because of the vulnerability and poor well-being of China’s factory workers [38]. One of the most remarkable results of China’s reform and opening-up policy since the early 1980s has been the emergence of massive rural-to-urban migration. According to a report by the National Bureau of Statistics of China, there were approximately 281.71 million rural migrant workers in 2017, among which 64.9% were male workers [39]. These rural migrant workers constitute not only the key actors in China’s rise as “the workshop of the world” but also the most marginalized group in contemporary China [40]. During China’s market-oriented reform, migrant workers, and in particular those who work in global factories, have become the “object-targets” for the state and global capitalists to achieve capital accumulation under the “global division of labor” [1]. For many “world factories” in China, how to manage the bodies of factory workers, to maximize their economic utility while diminishing their labor costs and consumption of welfare in the city, has been a crucial task for their survival under global capitalism [4,40]. This is particularly the case for Company A, an electronics manufacturing company. That said, evidence also shows that China’s factory workers are one of the most psychologically vulnerable groups in contemporary China, and suffer from occupational burnout, poor mental health and suicidal behaviors [38]. Factory workers’ well-being is highly influenced by the ways they work and are managed on the factory floor. In particular, male factory workers have a much higher rate (58.1/100,000) of suicide compared to women factory workers (30.2/100,000) [41]. Therefore, one of the research questions of this paper is: How do gender and especially the social construction of masculinity shape factory workers’ well-being at the workplace?

On the other hand, over the past three decades or so, China has undergone a nationwide religious revival, one manifestation of which is the “Christianity fever”. According to a report conducted by the Pew Research Centre [42], there were more than 58 million Protestants in China in 2010. The population of Protestants has increased over tenfold over a period of 30 years. Evangelization is particularly evident in the city that this paper focuses on, as it accommodates a large number of migrant workers. First, Protestant churches or other faith-based organizations have increasingly participated in offering both social and spiritual support for migrant workers. Since the suicide event of Company A, many Protestant churches have gradually switched their focus from urban middle-class onto the marginal migrant factory workers for evangelization and spiritual care. Second, large quantities of rural Christians migrated into the city in search of better livelihoods. In this sense, Christianity and Christian culture have significant influences among migrant-worker communities, and vice-versa (see [3]). This therefore leads to the second research question of this paper: How do religion and spirituality influence factory workers’ masculinity, as well as their psychological well-being and resilience?

### 3.2. Data Collection

The materials of this paper are based on a project that explores the regulatory politics of migrant laborers, as well as the religious beliefs and practices of migrant workers in China. In general, the project aims to examine the relation between religion and labor in the context China’s neoliberal transformation. One of the concerns of this project is to explore the gender politics of factory workers and its influences on well-being. Our data come from ethnography conducted from September 2017 to March 2018. The methods utilized in this study included participant observation and in-depth interviews. First, we closely followed factory workers’ everyday life practices in the factories, church and living spaces. Participant observation was used to gain an intimate familiarity with Christian factory workers. In particular, we participated in their Christian fellowships with the church’s permission in order to comprehend their understanding of masculinity. Participant observation not only provided important sources of data for this paper but also helped situate the analysis of interview data within specific social contexts [43] (see Section 3.3).

Second, we conducted interviews with various actors involved in China’s manufacturing factories (chief among them was Company A) and religious institutions. Interviews were conducted with 52 Christian factory workers and 8 church leaders. Among the 8 church leaders, 4 were senior and middle managers who were working or had worked at Company A. In total, the factory worker informants consisted of 32 males and 20 females, with ages varying from 16 to 60 (see Table 1). It needs to be noted that we were unable to recruit any women rank-and-file managers as our participants, although these women rank-and-file managers did exist in Company A. This was due to Company A’s masculine and militarized management system in which women were largely excluded from the positions of the rank-and-file manager that required them to be coercive and aggressive. This is also one of the limitations of this paper that we are unable to capture the experiences of women rank-and-file managers. All respondents were asked to sign a consent form before the interview, and were informed that all information linked to the participants will be treated in confidence and they could stop the interview at any time. Each interview lasted between 40 min and 3 h. All interviews were recorded and transcribed into a text of 616,978 Chinese characters. All the respondent names and the company name mentioned in the paper are pseudonyms.

### 3.3. Data Analysis and Interpretation

In this paper, we reconcile the Gioia Methodology [44] and Helliwell and Putnam’s theory of the social contexts of well-being [45] as the guidance for analyzing and interpreting interview data. The Gioia methodology is a form of grounded theory that helps scholars capture the data structure in qualitative research through the coding and conceptualization of the data [44]. It highlights the agency of the researcher in structuring and analyzing the original data. Gioia, Gorley and Hamilton note that: “the tandem reporting of both voices (informant and researcher) not only allowed a qualitatively rigorous demonstration of the links between the data and the induction of this new concept, sensegiving, but also allowed for the kind of insight that is the defining hallmark of high-quality qualitative research” [44] (p. 18). Specifically, Gioia methodology identifies different orders of data terms and themes for building data structure, which is especially useful in organizational and leadership studies [46]. On the other hand, Helliwell and Putnam argue that well-being is not an individual psychological state but rather a specific set of practices and beings that need to be understood within social and situated contexts [45]. Taken together, these two approaches help us to understand not only factory workers’ experiences but also the social structures that they are embedded in.

The interview data were coded with the qualitative data analysis tool NVivo 11 (QSR International, Melbourne, Australia). As a result, we generated 126 codes relevant to “masculinity, religion and well-being” from the raw data. Based on these 126 codes, we conducted 1st-order analysis, which tried “to adhere faithfully to informant terms” [44] (p. 20). This resulted in nine 1st-order concepts and four 2nd-order themes. An overview of the coding is presented below in Table 2.

## 4. Result

### 4.1. Contested and Hierarchical Masculinities and Their Influences on Well-Being within China’s Factories

At many manufacturing factories like Company A, a set of coercive and exploitative regulatory techniques, such as strict surveillance, standardized operations, monetary penalties, and even verbal abuse, are deployed to produce productive, docile workers on the factory floor. These governing techniques not only operate through “the modification of individual conduct” but also through processes of subjectivation that shape “how an individual acts upon himself” [47] (pp. 18–19). This is manifestly evident in Company A’s notoriously masculine and militarized management. Company A’s coercive management is characterized by the masculine and aggressive “culture” wherein managers often use verbal abuse and even physical punishment to devalue the esteem of lower-ranking assembly line workers to ensure workers’ absolute obedience to them. This masculine management style is not simply a common governing technique utilized by many other “sweatshops” in China, but also a peculiar “culture” evolved from the toxic leadership of Company A’s CEO and founder. It is argued that the CEO transplanted the bureaucratic system and masculine culture in the army into company management. For example, Long, a previous senior manager of Company A, as well as a Christian, told us his experience of working for the CEO:

The whole management system was designed by [the CEO]. He used to serve the military. You know, his personality, he is aggressive and dominating. He also required his subordinates acted like him. [He] doesn’t like others to ask him the rationale behind his orders and just needs you to be obedient, to follow and execute. If you can’t execute efficiently or make mistakes, he will scold you without any reservation. So, the atmosphere of Company A actually evolved from his own management philosophy.

The CEO’s management philosophy could also be captured in the saying “women are utilized as men while men are utilized as the non-human” among workers themselves. In essence, execution is the fundamental principle of Company A’s embrace of the global division of labor. Company A has a highly elaborate and hierarchical system through which orders and assignments are executed, and through which the coercive masculine culture can be performed by rank-and-file managers. At the level of the factory floor, a workshop is divided into different assembly lines, with 2–3 group leaders taking charge of the productivity assignments. Each assembly line is also divided into 3–4 sub-lines, with around 30–40 production workers and 1 sub-line leader per sub-line. The responsibility of the sub-line leader is to closely monitor the workers to ensure that the task is completed, and the production speed of workers is consistent and efficient. For assembly line workers, they are not allowed to engage in activities such as chatting, checking their smartphone or drinking water whilst working. When the rules are violated, or when workers make an operational mistake, line or group leaders would usually use verbal abuse to scold them. It is also common that managers use monetary penalties to threaten production workers to make them act obediently. Moreover, prior to the start of work, the line leader would give a lecture to all line members. As Zhicheng remarked, this lecture is more likely conducted to abuse workers rather than assign the tasks:

The morning lecture is something like that: the line leader normally assembles all members to assign the tasks and tell us what needs to be cautious. However, it usually ends up with scolding workers by picking their mistakes. They just want to devalue you.

Ironically, the ability to act coercively and use verbal abuse onto his subordinates is celebrated as a valuable personality trait that signals a manager’s capacity for controlling recalcitrant workers. Therefore, those who can act like the CEO are expected to have a better chance of promotion. In this sense, the CEO’s toxic leadership was successfully transformed into a masculine, militarized, and “Dog-Eat-Dog” culture and a normative management philosophy accepted by most managers at Company A. Under such atmospheres, when line leaders are pressured regarding orders and assignments from above, they often channel this pressure and negative emotions to lower-ranking workers at the expense of workers’ dignity and esteem. This unhealthy culture ultimately inflicts on the psychological well-being of workers by depriving their self-esteem. This is particularly the case for male workers, whose self-esteem, dignity and autonomy are key components of their masculinity. For example, the 35-year-old worker, Jinlong, told us that he felt no dignity in Company A:

I felt really oppressed. When you want to go to the toilet, you even need to ask the line leaders’ permission! They normally replied you impatiently: “wait a moment, I need to find someone to temporally replace you”. Then, I needed to hang on an “off-position card” on my neck to go to the toilet. I felt I had no dignity.

If the coerciveness and verbal abuse become hegemonic masculinity among Company A’s managers, then many male assembly line workers attempt to construct a form of protested masculinity that resists managers’ control by rebellious and aggressive practices. While facing the abuse from their supervisors, many male workers overtly use verbal violence to fight back and therefore to defend their masculinity. In particular, they would use *diaoren* (屌人) or *diaomao* (屌毛), a form of verbal violence that is prevalent in the workshop. *Diaoren* is a verb that means using dirty words to sneer and curse at others, while *diaomao* is a noun to describe low-status people. The word *diaomao* is usually used by male assembly line workers to depreciate themselves and thus construct and reinforce a working-class identity amongst their fellow low-status workers, or to devalue their supervisors.

Male production workers’ protested masculinity can also be illustrated by the frequent group conflicts between workers and Company A’s security guards. In order to enhance the militarized management, the CEO of Company A often recruited military veterans to be the factory’s security guards. In Company A, security guards were endowed with the rights to manage production workers in terms of enacting factory’s disciplines and rules. It is noteworthy that Company A’s first suicide was induced by the bullying and assault that the security guards inflicted on a young worker. Zhicheng, a 35-year-old worker explained to us how the conflicts occurred:

In Company A, it was common that the security guards abused workers with dirty language if the workers forgot to bring or swab the factory ID card. The security guard group do not belong to any [factory] floor or department, they are directly managed by the senior managers in Company A. So security guards and production workers are two confrontational groups in Company A. I remember there was even a group flight between workers and security guards at the canteen a few years ago. The cause of the conflict was that one worker insulted the security guards by calling them “watchdogs”. So they came to blows and many workers participated in. You know, there were just 4–5 security guards at the canteen and they were fiercely beaten up by the workers.

However, although the protested masculinity performed by male workers could be understood as a strategy to resist Company A’s hierarchical and disciplinary power, it is also harmful to the well-being of workers themselves. In fact, both security guards and production workers are low-ranked workers at Company A but they were placed into two confrontational positions. As Kazmierczyk suggests, mobbing and bullying more likely occur within employees/workers at a similar level and this kind of non-hierarchical mobbing has even greater detrimental effects on workers’ well-being [24].

Further, on the factory floor, the fear of being blamed and punished has created an unhealthy atmosphere of distrust among workers in which some workers tend to evade responsibility by passing the buck to each other once they are found to have made a mistake. It is common to see workers quarrelling with one another on the factory floor over the responsibility of the mistakes. In particular, women assembly line workers are the victims of both hierarchical and hegemonic masculinity from their supervisors and the protested masculinity from their male workmates. While many male production workers are verbally humiliated by their managers, they, in turn, construct their masculinity through claiming superiority over woman workers. As one female respondent recalled, it was common for male managers or production workers to overtly “talk dirty” and make sexual jokes and flirt with women workers in the workshops. In some cases, offensive language turned into sexual harassment, such as touching women’s bodies. However, this verbal violence and sexual harassment was viewed as natural by men. If the women tried to complain and resist, they would be viewed by male workers or managers as “too serious” and as someone who “can’t take a joke”. On the factory floor, there are therefore two forms of contested masculinities: the hierarchical masculinity among managers that encourages the exercise of power to control lower-ranking workers, and the protest masculinity [20] among male assembly line workers who utilize rebellious and confrontational practices to defend their dignity. Under such power relations, women workers suffer from the double oppressions from both hegemonic and patriarchal powers. Women workers were therefore more susceptible to unjust treatment in the workplace, as they would be viewed as more tolerant of exploitation by their managers. This can be illustrated by the story of a 23-year-old Christian girl, Xiaoli, who had been assigned by the line head to perform the same repetitive operation for two months because no male workers were willing take on this work:

Right after I was assigned to this assembly line, they asked me to assemble the phone screen, by just pressing the flexible printed circuit (FPC) into the phone model. I said: “OK, I have no objection”. But they asked me to do this every day, repeating the same action again and again with my two fingers. Other colleagues could switch to other operations but I wasn’t allowed to. So, I had pressed the FPCs for ten hours a day for one month and my fingers were injured already. After the first month, I talked to the line heads and asked them to let me shift to other positions, but they refused. The line head and assistant line head both asked me to continue. They (male lined leaders) said: “You do a good job on this. If I change to another one who is unfamiliar with this, he is more likely to destroy the FPCs, so more products will become scrap.”

**Figure 1:**
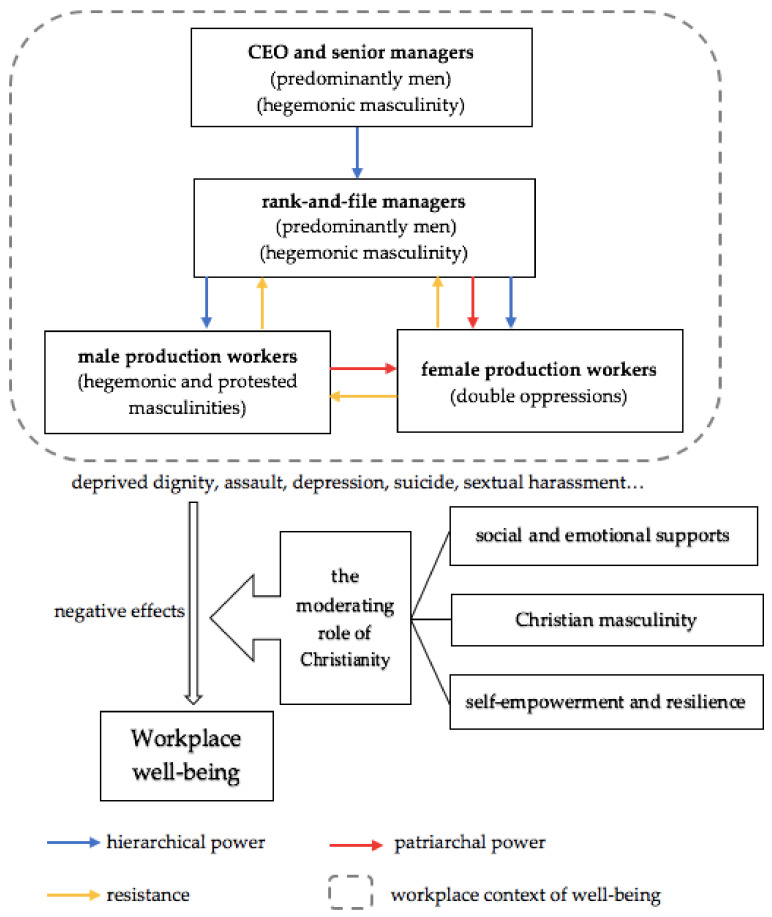
The model of masculinity, religion and workplace well-being. Note: CEO is the abbreviation of “Chief Executive Officer”.

As we show in Figure 1, compared with male production workers who were primarily the victims of hierarchical masculinity that deprived their dignity, women workers were actually experiencing multiple forms of oppression from their managers and male workmates. The suicide tragedy in Company A could be understood as an outburst of the long-term depression and exploitation under such a masculine and militarized management regime. Even though Company A has made efforts to improve workers’ working conditions, this masculine culture has been left largely unchallenged.

### 4.2. The Moderating Effects of Christianity on Masculinity and Workplace Well-Being

In this section, we elaborate on the moderating effects of Christianity on masculinity and workplace well-being. We suggest that Christianity can produce a Christian and morally superior manhood that enables factory workers to fend off and negotiate with the hierarchical and protested masculinities on the factory floor. This Christian masculinity therefore psychologically empowers them and enhances their resilience in the face of adversity and exploitation.

It is noteworthy that within Company A’s management system, a certain proportion of middle and senior managers appointed by its headquarters are Christians. Although these Christian managers would not overtly challenge the CEO’s masculine and militarized workplace culture, they normally refuse to accept coerciveness and aggressiveness as their own management philosophies, and attempt to blend them into a more humanist form in their “actually existing” management practices. Long and Liao were two of these Christian managers. For example, Long, a 40-year-old manager who used to work at Company A, offered his account of how a Christian manager should be:

In Company A, it is very easy to use abusive language to treat others. Gradually, we lost the ability to respect the colleagues and workmates around you. However, I don’t want to be that kind of supervisor, and I don’t want to be assimilated by this culture. This is against God’s teachings. When my subordinates made mistakes, I usually taught them what lessons can be learned rather than scolding them.

Different from Long who attempted to prevent himself from being assimilated by Company A’s culture, another 60-year-old manager, Liao, was more ambitious to transform Company A’s atmosphere and to revitalize the long-repressed humanism by introducing the Christian ethics of love and care into workers. In response to the suicide event, Liao established a house church right next to a factory plant of Company A in order to provide social and spiritual support for workers. In 2018, Liao’s Protestant church had more than 200 church members, most of whom were workers of Company A. The church provides these workers various forms of communal life (such as fellowship, dinners and choir) through which they can temporarily escape from their oppressive social realities. For example, Duo, a 35-year-old worker mentioned that the Christian fellowship offered him a comfort zone to confide in about his distress, even though he was ashamed to share his feelings of vulnerability elsewhere. This is particularly the case within Company A, where sharing the feelings of depression to workmates is at odds with the protested masculinity amongst working-class men. As Liao remarked, the lack of channels through which factory workers can pour out their troubles was one of the crucial factors that caused the suicide event. Instead, the church carves out an equal space built upon Christian ethics of brotherhood and love. It is common to see assembly-line workers and managers alike chat harmoniously with one another during the same congregation, which contrasts with the hierarchical and masculine culture of the factories.

For Liao, the Christian workers were an important force in transforming Company A’s masculine and abusive culture. Liao encouraged Christian workers to exhibit Christian virtues, such as caring for others and being helpful and trustworthy, so that non-Christian workers could feel the positive influence of Christianity. For male Christian workers, they have a different definition of masculinity beyond the hegemonic masculinity on the factory floor characterized by aggressiveness and verbal abuse. For example, Zhicheng was a 32-year-old Christian worker who had worked in Company A for 7 years. Zhicheng was promoted to a sub-line leader in 2017 because of his diligence and experience on the assembly line. In one fellowship, he shared his struggles of being a Christian rank-and-file manager, which was incompatible with Company A’s dominant culture of coercive management:

A girl (worker) said to me that she wanted to go to the toilet. We normally had a ten-minute break at 3:00 p.m. and now it was 2:45. So I politely responded, “It’s nearly 3 o’clock, you can take a break 15 min later”. The girl suddenly became extremely angry. It scared me, out of my expectation. I froze there, not knowing what I should say. Our line leader witnessed this scene… He said, “You shouldn’t let your workers deter you, you shouldn’t let her go to the toilet, it’s just 15 min”. I thought a while, said, “She is a girl, perhaps she was on her period and felt mood swings”. The line leader replied, “You shouldn’t act like that—consider the workers’ feelings. If you continue acting like this, being a *laohaoren* (one who tries to never offend anybody), you aren’t able to manage the workers”. I was thinking, if I acted coercively, it went against Bible’s teachings. The Bible teaches us to be benevolent and considerate of others.

Zhicheng offered an interpretation of what is Christian manhood: a Christian man should be considerate and caring of others, especially female colleagues. Zhicheng’s account of Christian masculinity was also influenced by the Christian teaching of male headship in the Bible. However, instead of interpreting male headship as men’s domination over women, Zhicheng told us that male headship meant men should take primary and more responsibilities in family and the workplace in comparison to women. Therefore, it is common to see that Christian male workers often voluntarily cover other workers’ shifts when their workmates are ill or exhausted, or undertake greater workloads to reduce the pressure on their workmates. Likewise, another 28-year-old Christian worker, Dong, understood Christian manhood as taking the sufferings by himself rather than transferring them to others:

In Company A, if a worker is often abused by his supervisors, he must have accumulated a lot of despair and hate. So, some workers chose to transferring their hate to others by abusing those who are weaker than them. For a Christian man, you should understand others’ sufferings. If a Christian is bullied by his supervisors, he should take the sufferings by himself rather than imposing your sufferings to others. It’s all God’s test on you.

In the above quotations, both Zhicheng and Dong pursued a Christian and moral manhood characterized by being benevolent, considerate and responsible. Although, this form of masculinity cannot challenge the hierarchical power from above, it nevertheless opens an alternative moral order within factory workers beyond the hegemonic and protested masculinities that encourage workers to turn against with each other. It is also noteworthy that Christian male workers exhibit stronger tolerance and resilience to exploitation and verbal abuse, given that the ontological conditions of social inequalities cannot be altered. For example, Dong considered the verbal abuse as “God’s test on you”. Many male Christian workers consider themselves as “God’s workers”, which means that they are working for God, not for the factory. It is through this alternative interpretation of work that the problem was deflected from this worldly labor relation per se. For example, a 27-year-old worker, Lijun, told us how he overcame the trouble of depression by converting to Christianity:

Now, I don’t need to care about any rules and regulations any longer, because I am working for God, not for the boss. The purpose of work is to glorify God. If you have such spiritual state, you can be peaceful regardless of how they treat you.

In this sense, by constituting an alternative and transcendent manhood, Christian male workers’ masculinity and dignity therefore detaches from the logics of winners and losers under the “Dog-Eat-Dog” culture at Company A. Moreover, this psychological self-empowerment also manifests in Christian migrant workers’ construction of a civil and morally superior manhood, which distinguishes them from rebellious and aggressive non-Christian workers. For example, many Christian workers believe that they are more civil and self-disciplined than non-Christian workers, who often engage in gambling and drinking. As Lijun further explained: “Because I believe in Jesus, I am different from them (non-Christian workers). I will never talk dirty or gamble, in contrast to what *waibangren* (foreigner, referring to non-Christian) often do”. Similarly, another 28-year-old male worker Jinlong considered self-discipline and obligations as valuable assets that distinguished him from other non-Christian male workers:

Because you are a Christian, your behaviors must be more civil than them (non-Christian workers). Your inner life has changed, you have to live out the image of Jesus—manage your temper and control your emotional impulse. I used to often argue with the line leaders when I was unhappy. But after I converted to Christianity, I started to follow God’s teachings and I tried not to complain. It is my duty to do the work that I should do. Once you realised this, you can attain inner peace.

Both Lijun and Jinlong actually assumed a higher ground in which they felt morally superior to non-Christians. In Chinese tradition, *wen* (literary) and *wu* (martial and physical) are considered two normative models of Chinese masculinity [48]. The former addresses a Confucian form of masculinity that emphasizes civility and self-discipline. In this sense, Christian factory workers’ construction of masculinity can be understood as a reconciliation of Christian and Chinese wen masculinities. This moral manhood therefore provides factory workers with a cultural strategy through which they can psychologically empower them.

## 5. Discussion

We have identified three forms of masculinities on the factory floor of Company A: (1) the hierarchical and hegemonic masculinity performed by managers who use verbal and physical violence to control workers; (2) the protested masculinity that male assembly workers utilize with rebellious and aggressive practices to defend their deprived dignity and masculinity; (3) Christian masculinity where Christian workers attempt to constitute a morally superior subject to fend off both hierarchical and protested masculinities and to reframe their experience of exploitation. A model of how these variegated masculinities influence workplace well-being is presented below in Figure 1.

As we see in Figure 1, Company A’s militarized management constitutes a toxic workplace that creates detrimental effects on workers’ well-being, such as depression, lack of dignity and even suicidal behavior. Since the suicide event in 2010, Company A has introduced a set of techniques to prevent workers from attempting suicide, which included providing psychological counseling, improving the working environments and wages, and reducing excessive working hours for workers. However, despite the reforms, the suicides at Company A continued. In other words, the Company’s masculine and militarized management system (see Figure 1) has been left substantively unchanged.

On the other hand, religious institutions and values provide a way to moderate and ameliorate workers’ psychological vulnerability inflicted by the coercive and masculine management. Religion’s moderating effects on well-being operate through creating a Christian masculinity and increasing workers endurance and resilience in the face of exploitation. Furthermore, Christianity provides the social and emotional supports for both male and female factory workers by offering them a relaxing and equal space to moderate the sense of unhappiness. These Christian spaces also foster a more equal and harmonious gender relationship that cuts against the patriarchal and heretical masculinity on the factory floor. However, this kind of self-empowerment cannot alter the material base of labor exploitation per se but rather reframes the ways factory workers make sense of exploitation. In this sense, the moderating effects of religion on workers’ well-being are conditioned by specific cultural and power relations.

## 6. Conclusions

This paper has explored the social construction of masculinities in the workplace, and its impacts on factory workers’ psychological well-being. Then, we explored the moderating effects of religion on the constructions of masculinity and well-being. This paper has two contributions to research on masculinity and health. First, we have developed Courtenay’s theory of masculinity and health [5] by bringing it into contextual focus through the workplace, and in particular China’s labor relations. The social construction of masculinity enriches our understanding of domination and labor exploitation in the workplace. This study shows that hegemonic masculinity has become a governing technique to manage workers and rank-and-file managers and specifically to produce docile and productive bodies of workers. In particular, the masculine management implicitly encourages lower-ranking workers to fight against one another and thereby turns them into atomized laboring subjects who cannot challenge the governing regime. Second, we have advanced research on religion and health [26,27,28] by offering a gendered understanding of health: religion enhances workers’ endurance and resilience by producing an alternative and Christian manhood that psychologically empowers them. Moreover, this paper also provides policy reference for improving factory workers’ working conditions and well-being in China. First, to improve workers’ well-being, it is not only the exploitative management of individual factories but also the global production system that needs to be reformed. International enterprises that expect contractors to increase productivity and cut costs, should also be held accountable for workers’ poor well-being. Second, the Chinese government should also allow more spaces for religious institutions or organizations to fill the gaps in social welfare left by the state.

However, the limitations of this paper are also noticeable. First, the paper primarily focuses on male factory workers’ experiences of male leadership, and women workers’ agency and coping strategies under the militarized and masculine management have not been fully examined. Second, it cannot reflect the dramatic transformations of China’s global factories (e.g., stricter implementation of labor law and the introduction of robots to replace workers) in the past four years and how these changes impact on workers’ well-being. Third, this paper cannot cover masculinities’ influence on well-being in other industry sectors and management models. Accordingly, future research can provide quantitative and comparative studies to understand how different models of management/leadership influence workers’ health and well-being. Further, research can also extend to examining more complex intersectional identities (e.g., Muslim men and homosexual men’s masculinities) and their relation to health.

## Figures and Tables

**Table 1:** Demographic information of participants.

Category	Sub-Category	Number
Gender	male	40
	female	20
Age	16–30	35
	30–40	15
	40–50	8
	50–60	2
Position	church leader	8
	senior and middle manager *	4
	rank-and-file manager	3
	production worker	49

* 4 of 8 church leaders were senior and middle managers.

**Table 2:** Masculinity, religion and well-being codes.

Examples of Illustrative Quote	1st-Order Concepts	2nd-Order Themes	Aggregate Dimensions
- “You aren’t allowed to leave you position for going to the toilet with line leaders’ permission.”	militarized management	toxic leadership and hierarchical masculinity’s influence on well-being	masculinities’ influence on well-being
- “diaoren”; “If the products pile up in front of you, the line leaders will immediately come to scold you.”	verbal abuse and bodily discipline		
- “It made us like robots, people with no personality. They very much emphasise discipline.”	deprived dignity and depression		
- “You can find quarrels between workers and their line leaders every day.”	rebellious practices	protested masculinity’s influence on well-being	
- “The line leaders prefer bullying the weak, so they often bully women workers.”	domination over women		
- “No matter how you dress, in a business suit or in ragged clothes, we should call each other brother and sister.”	church as a space of equality	social and emotional supports	the moderating role of religion in masculinities’ influence on well-being
- “I go to church because I love the atmosphere of this church, I can relax and feel free to talk with other brothers and sisters.”	church as a space of relax		
- “A Christian man should live out the image of Jesus, being considerate of their workmates.”	love and being considerate	Christian masculinity	
- “The man is the headship of the family. Men have been given the role as the head by God.”	male headship		
- “Now I realize that I am working for God.”	Christian interpretation of work	resilience and self-empowerment	
- “But I am not afraid of this any longer because God is with me now.”	endurance and tolerance		
